# Air evacuation under high-level biosafety containment: the aeromedical isolation team.

**DOI:** 10.3201/eid0502.990208

**Published:** 1999

**Authors:** G W Christopher, E M Eitzen

**Affiliations:** U.S. Army Medical Research Institute of Infectious Diseases, Fort Detrick, Maryland 21702-5011, USA.george_christopher@detrick.army.mil

## Abstract

Military contingency operations in tropical environments and potential use of biological weapons by adversaries may place troops at risk for potentially lethal contagious infections (e.g., viral hemorrhagic fevers, plague, and zoonotic poxvirus infections). Diagnosis and treatment of such infections would be expedited by evacuating a limited number of patients to a facility with containment laboratories. To safely evacuate such patients by military aircraft and minimize the risk for transmission to air crews, caregivers, and civilians, the U.S. Army Medical Research Institute of Infectious Diseases maintains an aeromedical isolation team. This rapid response team, which has worldwide airlift capability designed to evacuate and manage patients under high-level containment, also offers a portable containment laboratory, limited environmental decontamination, and specialized consultative expertise. This article also examines technical aspects of the team's equipment, training, capabilities, and deployments.


### Air Evacuation under High-Level Biosafety Containment: The Aeromedical Isolation Team[1]

**George W. Christopher and Edward M. Eitzen, Jr.**
U.S. Army Medical Research Institute of Infectious Diseases,
Fort Detrick, Maryland, USA

Military contingency operations in tropical environments and potential use of biological weapons by adversaries may place troops at risk for potentially lethal contagious infections (e.g., viral hemorrhagic fevers, plague, and zoonotic poxvirus infections). Diagnosis and treatment of such infections would be expedited by evacuating a limited number of patients to a facility with containment laboratories. To safely evacuate such patients by military aircraft and minimize the risk for transmission to air crews, caregivers, and civilians, the U.S. Army Medical Research Institute of Infectious Diseases maintains an aeromedical isolation team. This rapid response team, which has worldwide airlift capability designed to evacuate and manage patients under high-level containment, also offers a portable containment laboratory, limited environmental decontamination, and specialized consultative expertise. This article also examines technical aspects of the team's equipment, training, capabilities, and deployments.

Air evacuation of patients with potentially lethal, contagious infections poses unique challenges and risks to air crews and medical personnel. Evacuation of such patients is relevant to military contingency operations because troops may be placed at risk for hemorrhagic fevers and other infections during deployment to tropical environments or by adversaries' use of biological warfare agents.

Evacuation of patients to the U.S. Army Medical Research Institute of Infectious Diseases (USAMRIID) would afford the immediate availability of biosafety level 4 laboratories (designed for the study of pathogens requiring maximum biological containment for laboratory safety) and facilitate rapid diagnosis of diseases due to pathogens posing extraordinary laboratory safety hazards. Furthermore, USAMRIID has the only fixed patient-care suite in the world designed for medical care under maximum biological containment. To safely evacuate a limited number of patients to the containment-care suite and provide medical care while minimizing the risk for transmission to air crews, caregivers, and civilians, USAMRIID maintains an aeromedical isolation team (1-3).

Address for correspondence: George W. Christopher, USAMRIID, ATTN: MCMR-UIZ-T, 1425 Porter Street, Fort Detrick, MD, 21702-5011, USA; fax: 301-619-2312; e-mail: george_christopher@detrick.army.mil.

### The Aeromedical Isolation Team

The purpose of the isolation team is to safely transport patients with potentially lethal communicable diseases for which no effective vaccines, chemoprophylaxis, or specific therapies exist. These would include patients with an unknown disease pending identification of the pathogen, patients with viral hemorrhagic fevers (notably those due to filoviruses and arenaviruses), and those suspected of being affected by a biological attack (Table 1) (3). Etiologic diagnosis and medical care would be provided at USAMRIID.

USAMRIID can simultaneously deploy two teams, each consisting of one physician, one registered nurse, and four to six medics. Each team can transport and manage one patient. In addition, the team can deploy a portable containment laboratory with rapid diagnostic

[1]An earlier version of this paper was presented at the 84th Panel Symposium of the North Atlantic Treaty Organization Aerospace Medical Panel, Aeromedical Support Issues in Contingency Operations, Session VII, Control of Communicable Diseases, Rotterdam, The Netherlands, 1 October 1997.

Table 1. Infections and conditions requiring containment care during transport

| |
|---|
| Arenavirus infection |
| Argentine hemorrhagic fever (Junin virus) |
| Bolivian hemorrhagic fever (Machupo virus) |
| Brazilian hemorrhagic fever (Sabiá virus) |
| Lassa fever |
| Venezuelan hemorrhagic fever (Guanarito virus) |
| Bunyavirus infection |
| Congo-Crimean hemorrhagic fever |
| Filovirus infection |
| Ebola |
| Marburg |
| Orthopoxvirus infection |
| Monkeypox |
| Variola |
| Pneumonic plague until sputum cultures are negative |
| Any unknown, virulent, communicable disease pending diagnosis |
| Suspected biological-warfare–caused infection |

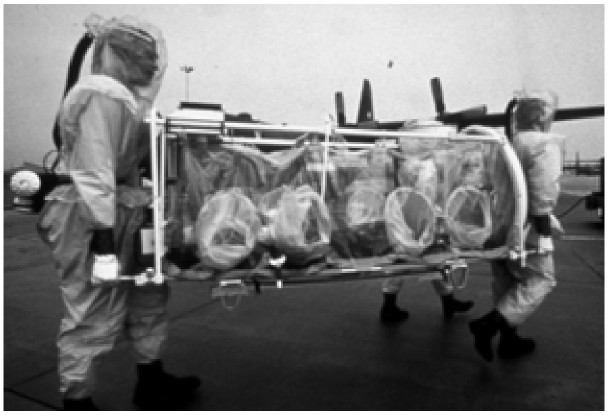

Figure 1: Aeromedical isolation team members in field-protective suits equipped with battery-powered HEPA-filtered respirators transporting the stretcher isolator, a light-weight unit designed for initial patient retrieval. The team trains on several types of military aircraft, including the C-130 transport shown in the background.

assays, including enzyme-linked immunosorbent assays and polymerase chain reaction (PCR), as well as standard clinical laboratory support, for all agents listed in Table 1 (in development: Machupo, Sabiá, and Guanarito viruses).

Deployable on rotary and fixed-wing military aircraft, the team conducts in-flight training and can deploy within 6 to 12 hours of notification. Although a military asset, the team has been mobilized for situations involving civilians. Requests may be forwarded through local and state health departments to the Centers for Disease Control and Prevention, through the Federal Emergency Management Agency, or through the Federal Bureau of Investigation and are then reviewed by the Directorate of Military Support. Evacuation of non-U.S. citizens from other countries to the United States would require coordination through the Department of State, Bureau of Political-Military Affairs.

### Biosafety Containment under Field Conditions

Maximum biological containment is designed to prevent transmission of highly hazardous pathogens and is accomplished in two steps. First, the health-care worker wears an impermeable suit consisting of a lightweight polyvinyl chloride (PVC) coverall, a separate hood, and vinyl boots (Figure 1). A HEPA-filtered respirator powered by a rechargeable battery supplies air under positive pressure for breathing and cooling. HEPA filters are certified to remove 99.7% of particles 0.03 µm to 3.0 µm diameter; each filter is tested with particulate aerosol challenge studies before delivery. Air enters at a rate of 170 L/min through an intake port near the top of the hood and exits through an exhaust valve at its base. Two-way radios permit communication between team members and patients. The suit and respirator ensemble has been tested by the manufacturer by particulate aerosol challenge and meets the standards of the National Institute of Occupational Safety and Health and the Occupational Safety and Health Administration for working in environments with respiratory hazards.

Second, the patient is isolated within a sealed container under negative air pressure maintained by a battery-powered HEPA-filtered ventilation system providing five air exchanges per hour (Figure 1). Two isolators are used: the stretcher isolator, a lightweight unit for initial patient retrieval (Figure 1), and the Vickers aircraft transport isolator (VATI), a larger unit for definitive transport and in-flight care (Figure 2; Table 2).

The design and construction of the isolators are similar to that of transparent flexible PVC isolators for gnotobiotic animals in biomedical research; the isolators were adapted both for in-patient and transport use (4-7). Challenge studies have demonstrated the efficacy of containing aerosolized T-2 bacteriophage during

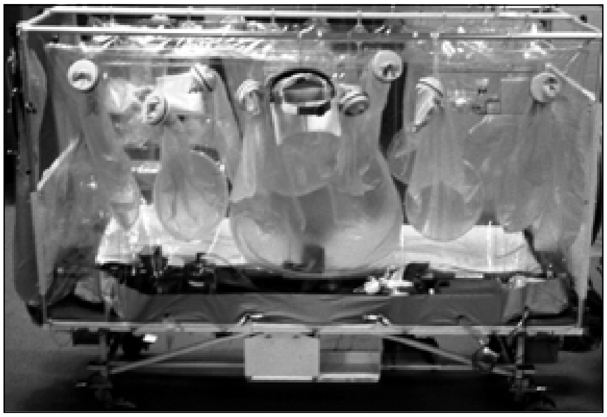

Figure 2: The Vickers aircraft transport isolator (VATI), designed for prolonged patient transportation and in-flight care.

Table 2. Dimensions of portable isolators

| Isolator | Length (cm) | Width (cm) | Height (cm) | Weight (kg) |
|---|---|---|---|---|
| Stretcher | 221 | 69 | 86 | 45 |
| Vickers aircraft transport | 221 | 91 | 152 | 112 |

both hypobaric and isobaric conditions (8). The HEPA filters are certified to remove 99.7% of all particles 0.3 µm to 3 µm in diameter. Isolators have been used to treat in-patients with suspected Ebola, Lassa, and Marburg hemorrhagic fevers (5,6). The utility and safety of these isolators for in-patient care have been questioned (9), and their use in hospitals is not recommended (10,11). However, transport isolators, the only available technical means of reliably maintaining airborne isolation in a military transport aircraft, have been successfully used for the aeromedical evacuation of patients with suspected Ebola fever (6) and suspected (7) and proven Lassa fever (9).

Both isolators feature transparent PVC envelopes suspended from metal frames by detachable plastic rings. Both envelopes include gloved sleeves, transfer and docking ports for patient entry, and transfer and supply ports for introducing supplies. Electrical current is supplied by rechargeable batteries or the aircraft electrical system. Both isolators can be equipped with portable oxygen tanks, intravenous fluids and tubing, medication, and portable defibrillators.

### Aeromedical Evacuation Process

The patient must be evaluated and stabilized before transport to ensure survival en route. Only patients likely to survive transport would be evacuated. The physiologic effects of altitude, effect of confinement on patient-care delivery, and psychologic effect of confinement within the isolator must be considered. Mechanical ventilation cannot be provided in the VATI, and suction capabilities are limited; therefore, acute respiratory failure and presence of gas trapped within closed body cavities that may pressurize at high altitudes (e.g., pneumothorax or intestinal gas due to ileus or bowel obstruction) contraindicate evacuation. Evacuation of patients with conditions requiring special in-flight management, e.g., hemodynamic instabilty and severe anemia (<2.5 million erythrocytes/cc or <7.0 g hemoglobin/100 ml) (12), may also be contraindicated.

The patient is placed inside the stretcher isolator and carried to a transfer point near the aircraft. There the stretcher isolator and team members are decontaminated with a 5% hypochlorite solution. During the decontamination procedure, the patient breathes portable oxygen from a mask, and the ventilation intake port is sealed to prevent chlorine gas from entering the isolator. The portals of the isolators are then connected with an airtight sleeve, and the patient is transferred to the VATI (Figure 3). The sleeve is clamped at two points before being heat-sealed and cut, maintaining air-tight seals throughout the transfer. The cut ends are decontaminated and covered with PVC seals, which are then attached to the isolators with

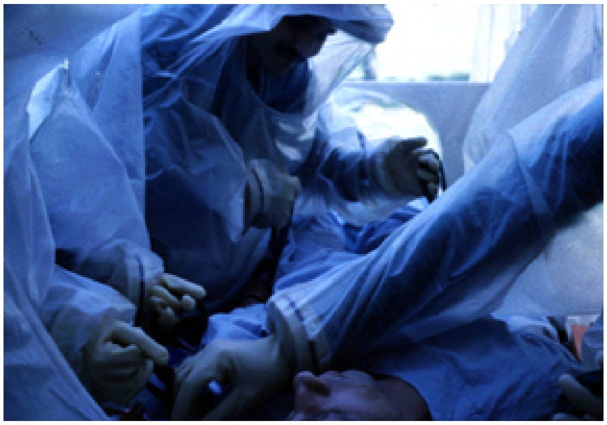

Figure 3: Patient's view of an aeromedical isolation team member providing care through a half-suit in the Vickers aircraft transport isolator (VATI).

pressure-sensitive tape. Both isolators are maintained under negative air pressure until decontaminated at USAMRIID. Equipment is removed, placed in bags, and returned to USAMRIID for decontamination of respirators and radios and disposal or decontamination of coveralls.

The patient is transported on standard military transport aircraft (C-130 or C-141), which maintain an internal cabin atmosphere equivalent to approximately 8,000 feet above sea level while at altitude (26,000 feet to 35,000 feet). This level of air pressure is considered adequate to protect commercial airline passengers (13) and results in an arterial blood hemoglobin oxygen saturation of approximately 90% in healthy persons. However, because the VATI maintains negative air pressure, the partial pressure of oxygen inside the VATI is lower than that of ambient atmosphere. This lower pressure would be hazardous for persons with respiratory failure or chronic obstructive pulmonary disease. In addition, rapid decompression could place the patient at further risk. Accordingly, the VATI is deployed with portable oxygen tanks, tubing, and masks capable of delivering 100% of needed oxygen.

Design features of the VATI that facilitate in-flight care include its larger size, additional glove ports, two half-suits, 12 cones at the base of the envelope for introducing wires and tubing, two sleeves for intravenous therapy, and two large pockets for placing waste supplies.

Diagnosis and therapy, which can be delivered in the VATI, include monitoring cardiac function, blood-pressure, and oxygen saturation of the blood; providing oxygen supplementation, intravenous therapy, and phlebotomy; and determining hemoglobin and hematocrit levels and serum electrolytes (by using a portable hand-held laboratory analyzer) (Figure 3). Because the use of glove ports limits manual dexterity, team members practice these skills on each other during on-ground and in-flight training exercises. Endotracheal intubation, manual ventilatory assistance with a bag and valve device, and cardiopulmonary resuscitation are practiced on mannequins in the isolators. To minimize the risk of puncturing the isolator, no glass bottles or instruments with rough or sharp edges are used. Phlebotomy is minimized, and a needleless intravenous system is used.

After arriving at USAMRIID, the patient is transferred from the VATI into the containment-care suite through a plastic sleeve connected to a port on an outside wall.

### Aeromedical Isolation Team Deployments

The first of several team deployments occurred during the October 1989 epizootic of Ebola hemorrhagic fever among cynomolgus monkeys (*Maccaca fascicularis*) imported from the Philippines and held at a primate quarantine facility in Reston, Virginia (14-16). Because Ebola virus had been isolated only in association with epidemics of human disease in Africa, which had death rates of 53% to 88%, potential transmission of Ebola to animal handlers in the facility and secondary transmission to other members of the community were of concern. Aeromedical isolation team and additional personnel from USAMRIID were deployed. Animal handlers were trained in the use of suits and respirators, containment methods, decontamination, and waste disposal; 450 monkeys were humanely euthanized; and team members obtained specimens of blood and tissue for histopathologic and virologic studies and sealed and decontaminated the facility by paraformaldehyde fumigation followed by conventional disinfectants (16).

Respiratory transmission was suggested by the epizootic spread among monkeys housed in separate cages (with no opportunity for physical contact [16]) and by subclinical human infections. Serologic evidence of recent Ebola infection developed in four of the five animal handlers; only one had percutaneous blood exposure. None became ill, which suggested that the epizootic strain was not virulent for humans (17), and none of the 42 USAMRIID personnel participating became infected. The Ebola isolates from infected primates represented a newly described strain, Ebola Reston, genetically and taxonomically distinct from related human pathogens identified in Africa.

In another episode, an aeromedical isolation team member was deployed to Linkoping, Sweden, in January 1990 to assist in implementing biosafety containment for a patient suspected of having a viral hemorrhagic fever after returning from eastern Africa (1).

During November 1995, the team was deployed with a senior medical advisor after construction workers at Wright-Patterson Air

Force Base, near Dayton, Ohio, uncovered a buried cache of biological munitions. Some of the munitions, produced during the U.S. offensive biological warfare program (1942–1969) (18), were intact, but most were perforated due to corrosion of the munition casings. The munitions were brought inside a bunker by the U.S. Army Technical Escort Unit and sampled inside the VATI. Samples of liquid bomb fill and adjacent soil samples were transported in sealed containers on ice packs in accordance with U.S. Department of Transportation regulations by military aircraft to USAMRIID, the Naval Medical Research Institute, Bethesda, Maryland, and the Armed Forces Institute of Pathology, Washington, D.C. The bomb fill contained nonviable gram-negative bacteria (identified as *Brucella suis* by strain-specific PCR); soil samples tested positive for *Brucella* DNA and antigens and cultures yielded normal commensal flora but no growth of *Brucella* sp. Background soil and groundwater tested negative for *Brucella* DNA and antigens. All munitions were drained, and the fill and casings were sterilized by autoclave before disposal. Documents later retrieved confirmed that the munitions were bomblets filled with *B. suis* and used at the base from June to October 1954 to train personnel in viability testing and handling of biological weapons. After training was completed, the munitions were heated in a ground portable heater with an ambient temperature of 104°C for 4 hours each of 2 days, with the temperatures of the innermost munitions reaching 70°C to 74°C, and then buried.

Although not deployed, the team was on alert during 1994 for a laboratory-acquired Sabiá virus infection (Brazilian hemorrhagic fever) at Yale University (19) and during the 1995 Ebola epidemic in the former Zaire.

Lt. Colonel Christopher is chief of the Containment Care Medicine Department, Operational Medicine Division, USAMRIID. His interests include medical care delivery under biological containment and the formulation of medical practice guidelines for the care of biological warfare casualties.

Colonel Eitzen is chief of the Operational Medicine Division, USAMRIID. He directs the division in multiple activities to enhance medical biological defense, including medical education programs; collaboration of basic scientific, medical, public health, and operational communities; and consultative expertise for military and other government agencies.

### References

1. Hill EE, McKee KT. Isolation and biocontainment of patients with highly hazardous infectious diseases. Journal of the U.S. Army Medical Department 1991;PB 8-91-1/2:10-4.
2. Wilson KE, Driscoll DM. Mobile high-containment isolation: a unique patient care modality. Am J Infect Control 1987;15:120-4.
3. Franz DR, Parrott CD, Takafuji ET. The U.S. Biological Warfare and Biological Defense Programs. Textbook of military medicine, part I. In: Sidell FR, Takafuji E, Franz DR, editors. Washington: TMM Publications; 1997. p. 425-36.
4. Trexler PC. An isolator system for the maintenance of aseptic environments. Lancet 1973;1:91-3.
5. Trexler PC, Edmond RED, Evans B. Negative-pressure plastic isolator for patients with dangerous infections. BMJ 1977;559-61.
6. Clausen L, Bothwell TH, Isaacson M, Koornhof HJ, Gear GHS, McMurdo J, et al. Isolation and handling of patients with dangerous infectious disease. S Afr Med J 1978;53:238-42.
7. Clayton CJ. Containment aircraft transit isolator. Aviated Space Environ Med 1979;50:1067-72.
8. Hutchinson JP, Gray J, Flewett TH, Emond RTD, Evans B, Trexler PC. The safety of the Trexler isolator as judged by some physical and biological criteria: a report of experimental work at two centres. The Journal of Hygiene 1978;81:311-9.
9. Fisher-Hoch SP, Price ME, Craven RB, Price FM, Forthall DN, Sasso DR, et al. Safe intensive-care management of a severe case of Lassa fever with simple barrier nursing techniques. Lancet 1985;2:1227-9.
10. Centers for Disease Control. Management of patients with suspected viral hemorrhagic fever. MMWR Morb Mortal Wkly Rep 1988;37:1-15.
11. Centers for Disease Control and Prevention. Update: management of patients with suspected viral hemorrhagic fever—United States. MMWR Morb Mortal Wkly Rep 1995;44:475-9.
12. Johnson A Jr. Treatise on aeromedical evacuation: I. Administration and some medical considerations. Aviated Space Environ Med 1977;48:546-9.
13. National Academy of Sciences. The airliner cabin environment-air quality and safety. Washington: National Academy Press; 1986. p. 8.
14. Centers for Disease Control. Ebola virus infection in imported primates—Virginia, 1989. MMWR Morb Mortal Wkly Rep 1989;38:831-9.
15. Jahrling PB, Geisbert TW, Dalgard DW, Johnson ED, Ksiazek TG, Hall WC, et al. Preliminary report: isolation of Ebola virus from monkeys imported to USA. Lancet 1990;335:502-5.
16. Dalgard DW, Hardy RJ, Pearson SL, Pucak GJ, Quander RV, Zack PM, et al. Combined simian hemorrhagic fever and Ebola virus infection in cynomolgus monkeys. Lab Anim Sci 1992;42:152-7.
17. Centers for Disease Control. Update: filovirus infections among persons with occupational exposure to nonhuman primates. MMWR Morb Mortal Wkly Rep1990;39:266-7.

18. U.S. Dept of the Army. U.S. Army Activity in the U.S. Biological Warfare Programs. Washington: U.S. Dept of the Army; 24 February 1977;2. Publication DTIC B193427 L.

19. Barry M, Russi M, Armstrong L, Geller D, Tesh R, Dembry L, et al. Brief report: treatment of a laboratory acquired Sabiá virus infection. N Engl J Med 1995;333:294-6.
